# Response time scores on a reflexive attention task predict a child's inattention score from a parent report

**DOI:** 10.1371/journal.pone.0190724

**Published:** 2018-01-12

**Authors:** Rebecca A. Lundwall, Jordan F. Sgro, Julia Fanger

**Affiliations:** 1 Psychology Department, Brigham Young University, Provo, Utah, United States of America; 2 Sociology Department, Brigham Young University, Provo, Utah, United States of America; McGill University Faculty of Medicine, CANADA

## Abstract

Compared to sustained attention, only a small proportion of studies examine reflexive attention as a component of everyday attention. Understanding the significance of reflexive attention to everyday attention may inform better treatments for attentional disorders. Children from a general population (recruited when they were from 9–16 years old) completed an exogenously-cued task measuring the extent to which attention is captured by peripheral cue-target conditions. Parents completed a questionnaire reporting their child’s day-to-day attention. A general linear model indicated that parent-rated *inattention* predicted the increase in response time over baseline when a bright cue preceded the target (whether it was valid or invalid) but not when a dim cue preceded the target. More attentive children had more pronounced response time increases from baseline. Our findings suggest a link between a basic measure of cognition (response time difference scores) and parent observations. The findings have implications for increased understanding of the role of reflexive attention in the everyday attention of children.

## Introduction

Understanding associations between parent-rated attentional problems in children and computer-based task performance might increase our understanding of various components of attention. This could lead to improved interventions for children with attention deficits. Because attention is an important precursor to learning [1, 2], attentional problems in children can affect academic performance and children’s willingness to stay in school [3]. An increased understanding of contributions to day-to-day inattention in children and adolescents has already made it possible to cater interventions to children with varying degrees of attention deficits, such as by using computer training programs [4, 5]. However, these studies refer to the association between sustained attention-task performance and day-to-day attention. There are comparatively few studies of reflexive attention as a component of day-to-day attention in children. If reflexive attention is an important component of day-to-day attention [6], then we are missing an important potential target for improving overall attention.

While the connection between performance on a reflexive attention task and the everyday behavior of children at school and at home may not be obvious, there are reasons to believe such a connection may exist. For example, it is logical that the ability to shift attention in order to efficiently process information plays a vital role in succeeding at any task that requires shifts in attention at school, work, and in relationships. In addition, several researchers have found that children diagnosed with attention deficit disorder have problems with reflexive attention tasks as well as with sustained attention tasks [7–9].

One way to study reflexive attention is to use a Posner [10] cueing task. These tasks measure automatic attentional shifts. This means that the shifts in attention induced by Posner cueing tasks are not willful or effortful decisions of the respondents, as commonly studied in sustained attention tasks. In particular, Posner [10] cueing tasks are used to study a participant’s ability to automatically disengage attention [11] or ignore information that is familiar or irrelevant. Differences between RTs to a baseline cue condition and an invalid cue condition (RT_dual cue_—RT_invalid cue_) are used to obtain the extra mental processing time (RT costs) associated with the mental steps of disengaging and moving attention [12, 13]. A great deal of research relies on comparing the RTs to targets that were preceded by invalid, valid, or no cues [14]. Thus, while we know that invalid cues typically induce RT costs in adults, RT costs and benefits may be different in children and may show different patterns of relationship with everyday attention in children.

Because parent ratings of every day attention and computer tasks both provide important information for understanding children’s behavior, it makes sense that these sources of information be considered together to predict the need for intervention with specific children. There is currently limited research on the association of reflexive attention tasks and parent reports of everyday attentional behavior, indicating a need for such studies. We anticipate that parent reports of child day-to-day attentional behavior will be associated with performance on a reflexive attention task such that children with poorer parent-rated attention will not benefit as much from peripheral cues as children with higher parent-rated attention.

## Materials and methods

### Participants

Participants were recruited after Institutional Review Board (IRB) approval from Rice University and the University of Wisconsin-Madison. Additional data analysis was performed under Institutional Review Board approval from Brigham Young University. Participants were recruited between May 2012 and May 2013 from a sample of previous infant participants as part of a larger study [15]. We invited all children for whom we could find a current address. Children were recruited if they were 9–16 years old at the time of the study. Of the 756 children invited to participate, 202 participated in an in-person visit to the Waisman Center on the campus of the University of Wisconsin-Madison. All work was carried out in accordance with the ethical standards of both universities and with the Declaration of Helsinki, sixth revision. The parents of participants signed consent forms and the children signed assent forms. Participants received $10 after data collection. Of the 202 original participants, we excluded six children. Two children were excluded because they had a serious neurological diagnosis and four children were excluded for being outliers on *impulsivity* and *inattention*, described below. This left 196 children with data for analysis. Of the 196 children in the analyzed data set, 98 (50%) were female (as reported by parents). The mean age was 12.96 years (range 10.58–16.55 years).

### Measures

#### Parents’ subjective reports of inattention, inhibition, and impulsivity

One of each child’s parents completed the MacArthur Health and Behavior Questionnaire, Parent Version or HBQ-P [16, 17], which has been modified to be developmentally appropriate for parents reporting on older children. The HBQ-P has been used in several other studies [18–20]. The current study used summary scores for inattention, which involved six items. Parents used a three-point Likert scale (0–2) to indicate agreement with statements about the distractibility, concentration, and organization of their children. Because impulsivity and inhibition are sometimes associated in the literature with ADHD [21, 22] and logically could be related to raw RT, both summary scores were included to ensure that these variables would not be related to the reflexive attention task while determining if inattention summary scores would be related. It was not expected that either impulsivity or inhibition would be associated with RT difference scores because they both appear to influence raw RT more than the *difference* in RT between single cue and baseline (dual cue) trials. Nevertheless, we decided it was important to verify this expectation.

Multiple studies have suggested that divergent validity is an important aspect of construct validity and is important when reporting overall findings [23, 24]. Both impulsivity and inhibition could be useful to demonstrate divergent validity for our task and its association with certain parent ratings. It would also be useful to show convergent validity for our task and its association with parent-rated inattention in children.

A continuous variable (provided by the HBQ-P) was used to describe the levels of inattention for each participant, meaning that the higher the inattention score, the more difficulties there were with attention as reported by the parent. Inattention on the HBQ-P consists of six items. Inhibition has three items. Impulsivity has nine items. The scales were scored as the sum of the ratings (0–2) of all associated items. Inattention scores had a possible range from 0 to 12. The observed range was 0 to 9. Eight-two parents endorsed no items and 114 parents endorsed at least one inattention item.

#### Laboratory assessment of reflexive attention

Children who participated in the study completed a peripheral cueing task. Participants were tested in a darkened room on a 381 x 305 mm monitor with a 60 Hz refresh rate. The experimenter adjusted the participant's’ chair height so that each participant was comfortable and viewed the display monitor from 57 cm. This distance from the eyes to the screen was maintained throughout the study using a chin rest. Displays on the monitor were controlled by E-Prime software (Psychology Software Tools, Sharpsburg, PA). Each participant was instructed to make a right or left key press, corresponding with the right or left location of the target but to avoid responding to the cues. Additionally, the participant was told that the cues did not reliably signal the location of the target that would follow and should be ignored as much as possible.

Participants completed a computer task featuring “friendly” earth rocket (cues) and alien spaceship (targets) shown either to the right or left of a central fixation point (+). Cues had an inner edge of 7.0 degrees from central fixation, and targets had an inner edge of 5.7 degrees from central fixation. Prior to the target appearing, one of three conditions could occur: no cues, one cue, or two cues. When the earth rocket cues appeared, they flashed briefly on for 67 msec and off for 87 msec, and then an alien spaceship target could appear (93% of trials had targets and 7% were catch trials). For one-cue trials, the cue could be valid (appearing on the same side as the subsequent target) or invalid (appearing contralateral to the subsequent target). Children were instructed to “hit” the target (by making a right or left key press depending on the location of the target) but to avoid responding to rockets (which are “friendly ships,” the cues).

**Fig 1:**
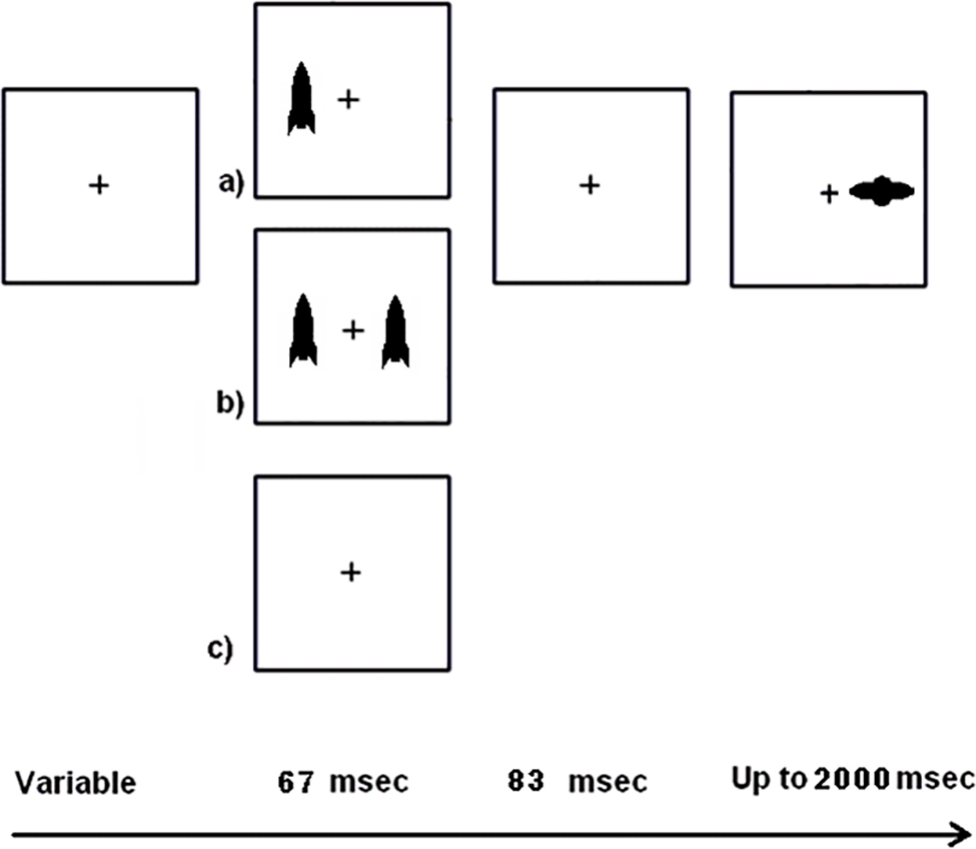
Schematic of child task. After the presentation of a fixation cross and variable delay, one, two, or no cues were presented and then disappeared. There was a brief delay and the target appeared on either the left or the right. Costs are associated with single cues that appeared opposite to where the target subsequently appeared.

In addition to being either valid or invalid, cues had two different saturations. They could be either unfaded (“bright”) or faded (“dim”), although targets were always bright. The use of two different cue saturations yielded seven primary measures: no cue, neutral dim, neutral bright, single bright valid, single bright invalid, single dim valid, and single dim invalid. Children were told to ignore the rockets (cues) because they did not provide information on the location of the targets (i.e., 50% validity). There were two testing periods lasting approximately eight minutes each with a five-minute break between. There were 24 trials of each condition intermixed and presented pseudo-randomly over the course of the task. In addition, there were 12 catch trials without a target in which respondents were told to withhold response. Only a respondent’s correct trials were used in the analyses. A schematic of the stimuli is shown in Fig 1. Benefits, and costs were calculated by subtracting RT to a given trial type from the appropriate baseline (e.g., either no cue trials for alerting effects or two cue trials for costs and benefits). See Table 1.

**Table 1:** The calculation of derived measures.

Derived Measure	Primary Measures Used in Calculation
Alert Bright	No Cue—Neutral Bright
Alert Dim	No Cue—Neutral Dim
Benefit Bright	Neutral Bright—Single Bright Valid
Benefit Dim	Neutral Dim—Single Dim Valid
Cost bright	Neutral Bright—Single Bright Invalid
Cost Dim	Neutral Dim—Single Dim Invalid

Note. The RT differences between the primary measures in the second column are used to calculate the derived measure in the first column.

### Statistical analyses

Because we planned to use general linear modeling, we checked to determine that our data met all assumptions. As mentioned above, we checked for univariate outliers and removed the data of four children who were outliers on impulsivity. The same four children were also outliers on inattention. We used Cook’s D to check for multivariate outliers. With a cutoff of .02 (4/196), there were 23 children with multivariate outlier data. However, the statistical decisions did not change when we excluded their data and so they were retained. All tests of Shapiro-Wilk normality were non-significant (*P*s > .13), indicating none of the HBQ-P score groups showed a significant departure from normality on the dependent variables. In addition, none of our variables showed multicollinearity (*R*s < .37). We used the GLM (general linear model) command in IBM’s SPSS, version 23 to determine the best outcome variable among *cost bright*, *cost dim*, *benefit bright*, and *benefit dim*. We attempted to associate parent-rated *inattention*, *inhibition*, and *impulsivity* scores with these outcome variables, expecting that inhibition and impulsivity would not associate. Child age and sex were used as covariates.

## Results

### Descriptive results

Parents rated their children on inattention (*M* = 1.68, *SD* = 2.06), inhibition (*M* = 1.21, *SD* = 1.20), and impulsivity (*M* = 2.37, *SD* = 2.55). Inattention and impulsivity were highly correlated (*r* = .72, *p* < .001) but inattention and inhibition (*r* = .08, *p* = .27) and impulsivity and inhibition (*r* = .09, *p* = .21) were not correlated. Because there is prior literature indicating a correlation between ADHD and impulsivity, and inhibition, and because multicollinearity is usually not an issue at *r* > .90 [25], we retained all three variables in the analysis. For all three variables, higher scores indicate more problematic behavior. Sex was significantly correlated with impulsivity (*r* = -.20, *p* = .01), indicating that boys were more impulsive. Sex was not correlated with inattention or inhibition (*P*s > .10). Age was likewise significantly correlated with impulsivity (*r* = -.23, *p* = .001), indicating that younger children were more impulsive, but age was not significantly associated with inattention or inhibition (*P*s > .36).

### Main analysis

We used a GLM to determine the effect of several predictors on the combined dependent variables. The dependent variables were cost bright, cost dim, benefit bright, and benefit dim. The predictors were inattention, inhibition, and impulsivity after controlling for age and sex. Neither impulsivity (*p* = .54) nor inhibition (*p* = .12) were significant for predicting the combined dependent variables. In addition, neither age (*p* = .17) nor sex (*p* = .80) were significant as covariates. Inattention was the only predictor that was significant (Pillai’s Trace = .06; *F*[4,187] = 2.72; *p* = .03, η_p_^2^ = .06) in predicting the combined dependent variables. Please note that this is a “medium” effect size according to Cohen [26]. In the ANOVA that accompanies GLM, we noted that benefit bright (*p* = .02) and cost bright (*p* = .01) were significant predictors of parent-rated inattention. In additional GLM analyses, we checked for interactions, but found none between age and inattention (*p* = .13) nor between sex and inattention (*p* = .99).

**Fig 2:**
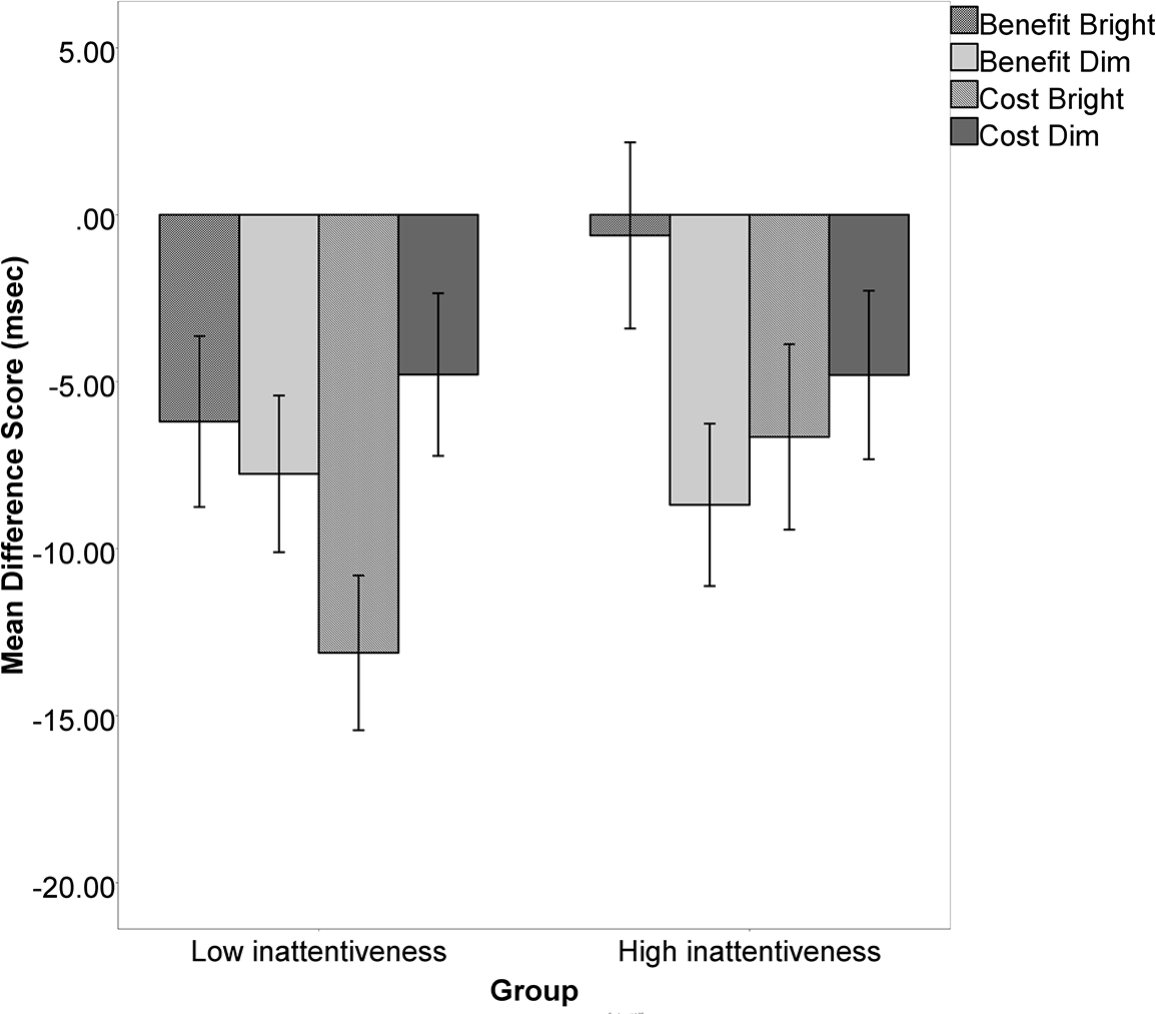
An illustration of the reflexive attention task scores if they are divided by low and high parent-rated inattention scores. In the post-hoc ANOVA, parent-rated inattention scores were significant predictors of continuous benefit bright and cost bright scores. Those children with lower parent-rated inattentiveness (better attention) had more pronounced slowing to bright cues whether they were valid or invalid.

These results indicate that lower inattention scores (fewer problems with inattention) were best predicted by responses to high saturation (“bright”) cues. As can be seen in Fig 2, when we group children in low versus high inattention score groups, children with lower inattention scores (whose parents endorsed fewer symptoms of inattention) had more pronounced negative RT difference scores (were slower than their own baselines) for benefit bright and cost dim on the computer task (also see Table 2). We explore these results further in the Discussion section.

**Table 2:** Benefit bright and cost bright mean difference scores by age group and inattentiveness group.

		BB (msec)		CB (msec)	
Age group (yrs)	Inattentiveness Group	Mean	SD	Mean	SD
10–5.8–11.80	low	-3.40	28.87	4.23	25.37
	high	7.39	33.31	3.31	27.87
11.81–13.66	low	-3.93	28.00	-11.93	27.42
	high	2.49	25.21	1.99	24.08
13.67–16.55	low	-0.18	22.18	-0.94	15.79
	high	-3.58	20.92	5.53	23.22

Note. Mean and SD values in this table control for age and sex. High inattentiveness is equivalent to poor attention.

## Discussion

### Main findings

In this study, we demonstrate that reflexive attention is an important contributor to everyday attention. This finding illustrates the link between a very basic function of the brain (information processing as measured by RT) and the HBQ-P inattention score, which is a much more general measure of attention obtained through the observations of a third party (the parent). The directionality of the effect over all ages is that children reported by their parents as having less inattentiveness had more pronounced costs following bright cues than children whose parents reported they were more inattentive, even when controlling for age and sex. However, this was not true for bright invalid cues for the youngest age group when the sample was divided into three groups (and age and sex were still controlled for). Instead, the low inattentiveness group did not experience costs. Therefore, peripheral bright stimuli may not be as distracting for younger children.

Our findings contradict previous results from Kean and Lambert [27] who found greater benefits following bright cues. In our study, there were essentially no benefits because valid and invalid cues were either not different or had larger (slower) RTs than their corresponding baseline conditions. However, our study may have different findings because Kean and Lambert [27] used adult participants and 80% predictable cues. Children’s responses are likely to be different from adults’ due to different distractibility [28]. Although age was not a significant predictor in our main analysis, there probably is some influence of development on reflexive attention when examining a larger age-span than we used [29]. The predictability used in Kean and Lambert [27] could have made bright cues worth attending to, but we discouraged voluntary attention to cues by making them unpredictable (50% probability that they would be valid) and by our instructions to “ignore them as much as possible.” While these differences make it difficult to determine if cue saturation is responsible for our different findings, our results are similar to those of Pratt, Hillis [30], who found that cues that were different from targets (our cues are vertical and our targets are horizontal; see Fig 1) tended to induce costs with bright cues. Although our bright cues were the same saturation as our target and Pratt, Hillis [30]’s bright cues had a higher luminance than their targets, the idea that bright cues induce costs, along with our testing ages, seem like the most logical explanations for the difference between our findings and those of previous researchers.

One possible implication of this study is that there may be additional targets for treatment of attentional deficits. Such targets may include neurotransmitters acting in the superior colliculus [31, 32] and parietal region [6]. Both these regions involve the cholinergic system, which has been noted elsewhere as a possible target for treatment [33]. Brain-behavior links suggests that the biological pathways that induce RT benefits and costs on a reflexive attention task might contribute to general day-to-day symptoms of inattention. This is consistent with reports from several researchers that reflexive attention is an important contributor to attentional deficits [7–9, 34]. The negative values shown in Fig 2 indicate slowing down when responding to a target following a single valid or invalid cue when compared to the dual cue (baseline) condition. The significant results suggest that the bright cues are less compelling for children as their inattention score (rated by parents) increases. Whether the bright cues were valid or invalid, they increased the RT cost (slowed responding relative to baseline) in children with lower inattentiveness (higher attention) scores. For children in the high inattentiveness group (less attentive as rated by their parents), benefits for bright valid cues did not differ from their baselines, and costs for bright invalid cues were not as large as for children in the low inattentiveness group.

In addition, it is important to consider the practical effect. Over the course of 180 trials (one minute on-task) the delays cost children about one extra second. Other researchers have found that peripheral cues like those we used can interrupt sustained attention [35]. In some situations, such a difference could be important. For example, stimuli peripheral to key content (e.g., in teaching slides) should not be bright because it will take some children about 6 seconds longer to process the information on such slides. Avoiding distracting content is especially important for developing engagement in students, since engagement can help a student’s academic success [36]. Although our study involves visual stimuli, it might also take attentive children longer to process auditory stimuli if there are auditory or visual (cross model) distractions. In addition, the older children in our study are beginning to drive; attentive children might be subject to longer braking times if there are bright stimuli in the periphery. Increased braking times do lead to more accidents, on average [37–39], although the drivers rated as attentive by their parents may be over confident because they think of themselves as attentive. In summary, our findings suggest that reflexive attention is worth investigating further for its practical implications.

### Limitations and future directions

One of our primary limitations in this study is with the lack of ethnic diversity. Our sample is 96% white consistent with the population from which we recruited. While these findings may generalize to all ethnic groups, we suggest using due caution and replicating this study with other ethnic groups. Several reports indicate that ADHD diagnosis varies with ethnic group [40, 41]. The cause of the variation in prevalence could be due to actual differences in attention or to parent and medical professional bias in who seeks or obtains a diagnosis [42]. Because the computer task does not suffer from the same risks for bias in presentation or human diagnosis, it would also be interesting to compare results between ethnic groups to ascertain the effects that culture may have on attentional diagnoses.

A further limitation of this study is that our age range is higher than appears ideal to identify and begin working with children with attentional deficits. Intervention should generally begin as early as possible before a child begins to experience trouble in school. A new study could potentially replicate the current study but use a younger population to determine whether or not benefit bright and cost bright are related to inattention as well in younger children.

While some researchers have proposed that parent reports of psychological symptoms may not be as reliable as child self-reports [43] or may be biased by the parents own psychopathology [44, 45], this is not a limitation of the current study because the parent-rated behaviors were related to an external criterion, computer task performance. In addition, multiple studies have found that parent-reports are more accurate under certain circumstances that apply to this study [46, 47]. In particular, Teye and Peaslee [48] found that younger and lower performing children are more likely to report inaccurately, again suggesting the need for parent reports. For all these reasons, it makes sense that researchers consider the reports of parents about their children’s behavior when assessing behaviors that could potentially contribute to the children having better educational outcomes.

## Conclusion

Here we have shown that parent insight into their child’s attention-related behaviors is associated with basic RT difference scores. Inattention scores were associated with the extra time it takes to respond to targets following bright cues. Such increased RT indicate that children with fewer inattentive behaviors (as rated by parents) are reflexively attending to cues they are told to ignore, and this is especially true for bright cues. The same is not true for children with more inattentive behaviors. Because the parent-rated behaviors reflect day-to-day observations a parent might be expected to have expertise about, this association suggests that a more foundational cognitive process might be influencing everyday child behaviors that the parent might notice. This was true even in a sample of children from the general population who were not selected for any attentional problems.
